# Pharmacist-Prescribed Hormonal Contraception: A Survey of Perceptions of Georgia Community Pharmacists and Non-Community Pharmacists

**DOI:** 10.3390/pharmacy12050156

**Published:** 2024-10-18

**Authors:** Rebecca H. Stone, Megha D. Patel, Lara L. Beene

**Affiliations:** College of Pharmacy, University of Georgia, Athens, GA 30602, USA

**Keywords:** hormonal contraception, pharmacist, pharmacy access, pharmacist-prescribed

## Abstract

Pharmacist-prescribed hormonal contraception (HC) is supported by a majority of pharmacists and pharmacy students; however, few studies have evaluated perceptions of non-community pharmacists, or differences in geographic areas. The primary objective of this study is to assess differences between community and non-community pharmacists in perceptions of pharmacist-prescribing HC in Georgia, a state that does not currently permit this practice. Secondary objectives include assessment of community pharmacist interest in prescribing HC, and differences in perceptions between pharmacists in metropolitan and nonmetropolitan areas. A survey was emailed in early 2022 to 2592 Georgia pharmacists, with Likert questions assessing interest, perceptions, comfort, and perceived barriers regarding pharmacist-prescribed HC. Chi square testing identified differences between groups. The completed survey response rate was 11.8%. Regardless of practice site, a majority agreed that pharmacists are well trained to prescribe HC (community 61.8% vs. non-community 68.1%, *p* = 0.25) and provision of HC services is within pharmacists’ scope (community 73.6% vs. non-community 74.2%, *p* = 0.90). Overall, metropolitan and nonmetropolitan community pharmacist perceptions were similar; however, more metropolitan pharmacists believed pharmacists are well trained to prescribe HC (66.7% vs. 48.7%, *p* = 0.049) and that it is within their scope of practice (78.1% vs. 61.5%, *p* = 0.045). In summary, the majority of pharmacists, regardless of practice type, believe that pharmacists are prepared to prescribe HC and that it is a part of pharmacists’ professional scope of practice.

## 1. Introduction

In 2017, 41% of pregnancies in Georgia were wanted later or unwanted [1]. Pharmacist-prescribed self-adminstered hormonal contraception (HC) is one strategy that has been implemented in an effort to improve patient access and decrease unintended pregnancy rates in the United States. In 2016, California and Oregon successfully implemented pharmacist-prescribed, self-administered hormonal contraception without a physician’s prescription. Currently, more than half of the states in the US have approved this expanded scope of practice [2]. This trend will likely continue, with more states allowing pharmacists to prescribe self-administered hormonal contraception. However, Georgia does not currently permit this expanded scope of practice [2].

Pharmacist-prescribed contraception is authorized under individual state protocols and/or regulations, and typically includes hormonal contraceptive pills, transdermal patches, vaginal rings, and in some states, medroxyprogesterone injections and/or oral emergency contraception [2]. Pharmacist training requirements vary by state, with most states requiring a program through the state Board of Pharmacy or Department of Health, but some states have integrated the required training into the curriculum of pharmacy schools in that state and do not require additional training [2]. Pharmacist-prescribed contraception regulations follow clinical guidance found in the United States Medical Eligibility Criteria [3] and Select Practice Recommendations for Contraceptive Use [4]. Requirements include a blood pressure check and a patient self-screening tool to assess for potential pregnancy and/or medical contraindications.

Multiple studies have assessed patient, pharmacist, and pharmacy student attitudes and interest in pharmacist-prescribed HC, and much of this data have been analyzed in a systematic review [5,6,7,8,9,10]. Data indicate that a majority in all three of these groups support this expanded scope of practice and intend to participate if available in their state. Additionally, data indicate that most pharmacy students view HC prescribing as part of their job description, regardless of whether or not it is approved in their state [9]. Contemporary national data also demonstrate that regardless of state approval, community pharmacists from across the United States support pharmacist-prescribed HC [8,10].

To date, few studies have assessed how pharmacists working in both community and non-community practice settings view this expanded scope of practice. One 2018 study conducted in a single state provides some insight, but it is unclear if prescribing HC is now viewed as a standard part of the pharmacist’s role by the profession as a whole, including pharmacists working in non-community practice settings [11]. Reporting the viewpoint of pharmacists as a whole, regardless of practice type, may help educators and lawmakers better understand and facilitate the contemporary pharmacist scope of practice, particularly in states where this authority has not yet been approved.

Studies evaluating oral emergency contraceptive access in Georgia have found differences in pharmacy access between metropolitan and nonmetropolitan areas for these medications [12]. However, it is unknown if there are differences between metropolitan and nonmetropolitan areas regarding pharmacist interest and attitudes in hormonal contraception prescribing, another aspect of reproductive health. Identifying facilitators and barriers to models that improve access to care is important because identified disparities may be addressed with interventions such as targeted education or outreach services.

This study’s primary objective is to assess differences between Georgia community and non-community pharmacists in their perceptions of pharmacist preparedness and scope of practice regarding HC prescribing. The secondary objective is to assess community pharmacist interest in providing this service in Georgia, and to compare differences in pharmacist interest, perceptions, comfort, and perceived barriers between metropolitan and nonmetropolitan counties.

## 2. Materials and Methods

### 2.1. Study Design and Participant Recruitment

This study was deemed exempt by the University of Georgia’s Institutional Review Board. The Georgia State Board of pharmacy is unable to provide a contact list for licensed Georgia pharmacists, so this voluntary online survey was emailed via Redcap to all pharmacist members in two professional organizations, the Georgia Pharmacist Association and Georgia Society of Health-System Pharmacists (n = 2592). The survey was emailed in mid-March of 2022 and survey responses were accepted for 1 week.

### 2.2. Data Collection and Measures

The survey was developed based on previously published research [6,8], took approximately 10 to 15 min to complete, and was administered entirely online. The survey collected demographic information (5 items), perceptions regarding pharmacist-prescribed HC if it were permitted in the state of Georgia (1 item), perceived barriers regarding pharmacist-prescribed HC (2 items), and desired education (1 item). Five-point Likert-style questions (1 = strongly agree to 5 = strongly disagree) assessed pharmacists’ interest (1 item), perceptions (6 items), comfort (6 items), and 3-point Likert-style questions (1 = very confident to 3 = not at all confident) assessed clinical confidence (2 items). The full survey is available to view in the Supplementary Materials, Figure S1. Responses were included in data analysis if they were completed by Georgia pharmacists with an active license, as verified by the State Board of Pharmacy online license lookup tool. All survey participants received a “$15 USD Amazon gift card via email for survey completion.

Pharmacists were categorized as community pharmacists if they worked full- or part-time in a community pharmacy. Pharmacists were categorized as non-community if they worked in other practice settings, including hospital, ambulatory care clinics, managed care, industry, or other. Respondents were classified by metropolitan or nonmetropolitan areas using the 2013 National Center for Health Statistics (NCHS) Urban-Rural Classification Scheme in the different Georgia counties [13].

### 2.3. Data Analysis

Descriptive statistics were used and reported as percentages based on survey results. Likert responses were compared across pharmacist practice area (community and non-community) and geographic area (metropolitan and nonmetropolitan). Chi square testing identified differences between groups. We used an alpha level of 0.05 for all statistical tests.

## 3. Results

Of the 2592 pharmacists who received the survey, 454 responded. Of these, 19 responses were excluded for being out of state, 7 had an unverifiable license number, and 121 had an email address that did not correspond with the reported license number. Three hundred and seven pharmacists with a verified active Georgia license completed the survey, for a response rate of 11.8%. Out of the 307 respondents, 163 (53.1%) identified as non-community pharmacists. The remaining 144 (46.9%) identified as community pharmacists, with 51 (35.4%) practicing in an independent pharmacy and 93 (64.6%) practicing in a chain pharmacy setting. There were demographic differences between community and non-community pharmacist groups in reported gender, geographic region, and age. See Table 1.

### 3.1. Community vs. Non-Community Pharmacist Perspectives

**Figure 1:**
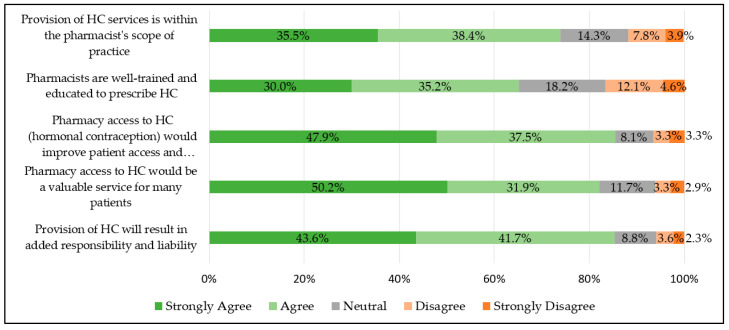
Pharmacist perceptions of hormonal contraception prescribing (n = 307).

Of the 307 pharmacists that responded to the survey, a majority “strongly agree” or “agree” that provision of HC services is within pharmacists’ scope of practice (total 73.9%; community 73.6% vs. non-community 74.2%, *p* = 0.90) and that pharmacists are well-trained and educated to prescribe HC (total 65.1%; community 61.8% vs. non-community 68.1%, *p* = 0.25). See Figure 1. A majority also “strongly agree” or “agree” that pharmacy access would improve patient access and adherence (total 85.3%; community 81.3% vs. non-community 89%, *p* = 0.06) and is a valuable service for many patients (total 82.1%; community 78.5% vs. non-community 85.3%, *p* = 0.12). Lastly, the majority of respondents “strongly agree” or “agree” with the statement that provision of HC will result in added responsibility and liability (e.g., requiring malpractice insurance) (total 85.3%; community 82.6% vs. non-community 87.7%, *p* = 0.21). There were no statistically significant differences in perceptions of hormonal contraception prescribing between community and non-community pharmacist responses.

### 3.2. Community Pharmacist Perspectives: Metropolitan vs. Nonmetropolitan

Of the 144 community pharmacists that completed the survey, regardless of metropolitan or nonmetropolitan geographic location, most “strongly agree” or “agree” that pharmacy access to HC would be a valuable service (total 78.5%; metropolitan 81.9% vs. nonmetropolitan 69.2%, *p* = 0.10) and improve patient access and adherence (total 81.3%; metropolitan 83.8% vs. nonmetropolitan 74.4%, *p* = 0.2), and that provision of HC will result in added responsibility and liability (total 82.6%; metropolitan 84.8% vs. 76.9% nonmetropolitan, *p* = 0.27). Metropolitan pharmacists were more likely than nonmetropolitan pharmacists to “strongly agree” or “agree” that pharmacists are well trained and educated to prescribe HC (total 61.8%; metropolitan 66.7% vs. nonmetropolitan 48.7%, *p* = 0.049), and that provision of HC services is within their scope of practice (total 73.6%; metropolitan 78.1% vs. nonmetropolitan 61.5%, *p* = 0.045).

### 3.3. Community Pharmacist Interest and Comfort: Metropolitan vs. Nonmetropolitan

**Figure 2:**
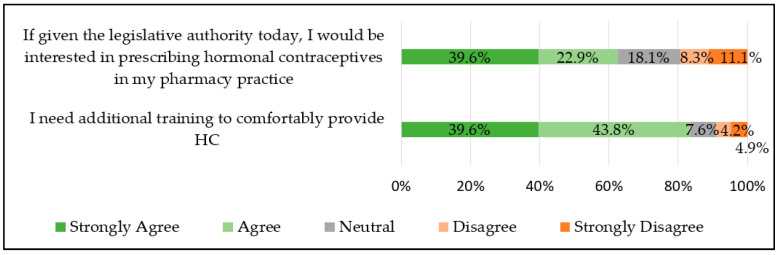
Community pharmacist interest in and comfort with prescribing hormonal contraception (n = 144).

The majority of community pharmacists “strongly agree” or “agree” that they would be personally interested in prescribing HC if given the legislative authority (total 62.5%; metropolitan 62.9% vs. nonmetropolitan 61.5%, *p* = 0.86). Most also “strongly agree” or “agree” that they would like additional training (total 83.3%; metropolitan 85.7% vs. nonmetropolitan 76.9%, *p* = 0.21). See Figure 2.

**Figure 3:**
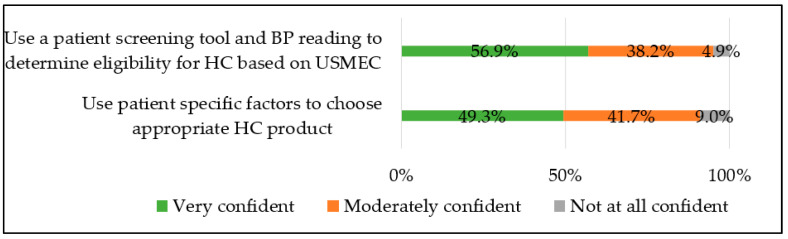
Community pharmacist confidence level for prescribing hormonal contraception (n = 144).

A majority of community pharmacists reported being “very” or “moderately confident” in using a patient screening tool and carrying out blood pressure reading to determine eligibility for HC (total 95.1%; metropolitan 96.1% vs. nonmetropolitan 92.3%, *p* = 0.5), and using patient-specific factors (i.e., past medical history, medication history, preferences) to choose an appropriate hormonal contraceptive (total 91%; metropolitan 93.3% vs. nonmetropolitan 84.6%, *p* = 0.24). See Figure 3.

**Figure 4:**
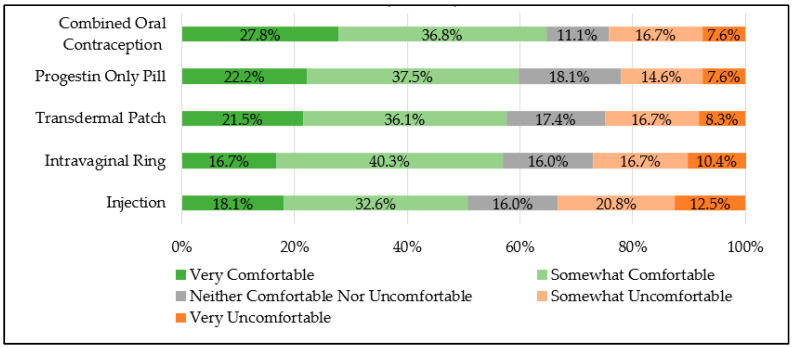
Community pharmacist comfort level in prescribing hormonal contraception (n = 144).

Regardless of metropolitan or nonmetropolitan location, a majority of community pharmacists reported being “very” or “somewhat comfortable” with counseling on all contraceptive products, including combined oral contraception (total 64.6%; metropolitan 62.9% vs. nonmetropolitan 69.2%, *p* = 0.48), progestin-only pills (total 59.7%; metropolitan 60.0% vs. nonmetropolitan 59.0%, *p* = 0.91), transdermal patches (total 57.6%; metropolitan 61.0% vs. nonmetropolitan 48.7%, *p* = 0.19), intra-vaginal rings (total 56.9%; metropolitan 61.0% vs. nonmetropolitan 46.2%, *p* = 0.11), and injections (total 50.7%; metropolitan 52.4% vs. nonmetropolitan 46.2%, *p* = 0.51). See Figure 4.

### 3.4. Community Pharmacist Educational Needs: Metropolitan vs. Nonmetropolitan

When asked about educational needs, there were no statistically significant differences between metropolitan and nonmetropolitan community pharmacists. See Table 2.

### 3.5. Community-Pharmacist-Reported Concerns/Potential Barriers: Metropolitan vs. Nonmetropolitan

There were no significant differences in the potential barriers identified between metropolitan and nonmetropolitan community pharmacists. See Table 3.

## 4. Discussion

A majority of Georgia pharmacists, both in community and non-community practice, believe that prescribing HC is within pharmacists’ scope of practice (74%) and that pharmacists are well trained and educated for this role (65%). Previously published national and state-level data also clearly demonstrate that community pharmacists believe prescribing HC is within their scope of practice and pharmacists are adequately prepared for this role; however, most of the literature does not report the perspectives of non-community pharmacists and is therefore limited in its assessment of the profession as a whole [5,6,7,8,9,10]. This study demonstrates that Georgia pharmacists as a whole view prescribing HC as part of the pharmacist scope of practice, despite lack of approval in their own state.

To the authors’ knowledge, one other study assesses both community and non-community pharmacist perspectives. It was conducted in North Carolina in 2018 when pharmacist-prescribed HC was not yet authorized, although this expanded scope was ultimately granted in 2022 [11]. Over half of all pharmacists agreed that prescribing HC allows pharmacists to practice at a higher level, rural areas would benefit from pharmacist prescribed HC, and increased access to HC is an important public health issue. Surprisingly, data indicated that non-community pharmacists were more likely than community pharmacists to agree with the aforementioned statements regarding pharmacist-prescribed HC [11].

This Georgia-based survey found, similar to North Carolina pharmacists, that a majority of pharmacists “agree” or “strongly agree” that pharmacy access would be a valuable service for many patients (82%) and that pharmacy access would improve patient access and adherence (85%). Although there were many similarities between the two survey studies, there were a few notable differences. Approximately 65% of Georgia pharmacists “agree” or “strongly agree” that pharmacists are well trained/educated to prescribe HC, compared to less than 50% of North Carolina pharmacists [11]. This may be in part because the North Carolina study was conducted 3 years earlier, and pharmacist confidence in the doctor of pharmacy education and training for this activity may have contiued to increase over this time.

Taken together, these two studies indicate that pharmacists, regardless of practice type or state regulations, now perceive pharmacist-prescribed, self-administered hormonal contraception as an established activity within the community pharmacy scope of practice. Regardless of practice site, pharmacists believe it is a valuable service for patients and will improve patient access and adherence.

When comparing the perspectives of community pharmacists in metropolitan and nonmetropolitan areas, there were relatively few differences. Regardless of location in Georgia, a majority of community pharmacists (62%) are interested in prescribing hormonal contraception. This is similar to other national contemporary studies that indicate 65% of pharmacists are interested in providing direct access to hormonal contraception [8]. Community pharmacists in metropolitan and nonmetropolitan regions believe pharmacist-prescribed HC is good for patient care.

Additionally, most Georgia community pharmacists indicated that they felt confident using a patient self-screening tool (assessing for potential pregnancy and/or medical contraindications), and BP readings to determine eligibility for HC based on the United States Medical Eligibility Criteria for Contraceptive Use (USMEC) and the United States Selected Practice Recommendations for Contraceptive Use (USSPR), which provide clinical recommendations and standards of care for contraceptive use [3,4]. Most also felt confident using patient-specific factors to choose an appropriate HC product. As other national studies have also identified, although most believe pharmacists are well-prepared, over 80% still desire additional training prior to offering this service [14]. This may be related to the fact that although pharmacists complete contraception training in their degree program, years may have passed since they received any formal education in this area, and they may desire continuing education to stay abreast of current trends and practice updates. This point is often addressed by the state board of pharmacy by requiring additional certification and/or continuing education in this area, typically a 4 to 6 h accredited course, in order to provide this service.

However, more community pharmacists in metropolitan areas believed that pharmacists are well trained and educated to prescribe HC and that it is within their scope of practice. Another study in Oregon found that pharmacists in urban areas were more likely to indicate that they were planning to offer pharmacist-prescribed contraception [15]. In contrast, perceptions of pharmacist-prescribed HC in other states have not demonstrated a difference between rural and urban areas. One study conducted in New Mexico following implementation of pharmacist prescribing found that rural pharmacists were as likely as their urban counterparts to prescribe hormonal contraception [16]. Another conducted in North Carolina found attitudes regarding pharmacist-prescribed hormonal contraception did not significantly differ by geographic region [17]. Although the objectives of this Georgia-based survey did not specifically set out to evaluate gender, the authors noted that gender may have potentially contributed to this difference in this study, as 46% of participants in nonmetropolitan areas were male compared to only 29% in metropolitan areas.

Since the completion of this study, the FDA has approved an over-the-counter progestin-only contraceptive pill [18]. Unlike pharmacist-prescribed hormonal contraception, which is regulated at the state level, this change allows uniform over-the-counter access to this specific contraceptive method across the United States. The results of this study may be applicable to the OTC progestin-only pill in that pharmacists believe pharmacy access to hormonal contraception is a valuable service for patients, and metropolitan pharmacists may initially feel better prepared to recommend or provide counseling for this product.

Similar to other national studies, pharmacist-identified barriers included concerns regarding lack of access to medical records, missing preventative care, time constraints, inadequate compensation, increased liability, resistance from physicians, workflow disturbances, and gaps in contraceptive knowledge [5,6,7,8,9,11]. However, the continued expansion of this service across the United States demonstrates that the profession of pharmacy is able to mitigate or overcome these barriers [2,19].

Limitations of this study include its small size and analysis of data from just one state. This pilot study recruited a convenience sample through state pharmacy organizations, and we did not identify a specific sample size to power our analysis. There were demographic differences between the community and non-community pharmacy groups in this convenience sample. The results of this voluntary study may have been subject to selection bias and nonresponse bias. This survey did not collect participant names but did collect pharmacist license numbers and email addresses for gift card verification. Supplying this identifiable information may have increased the likelihood of social desirability bias in participant responses.

## 5. Conclusions

This study found that in a state without pharmacist prescribing, the vast majority of pharmacists, regardless of practice site, believe that (1) pharmacists are adequately educated to prescribe HC, (2) prescribing HC is part of pharmacists’ professional scope of practice, and (3) pharmacists are interested in prescribing HC. Metropolitan pharmacists were more likely to believe that (1) pharmacists are well trained and educated to prescribe HC and (2) it is within their scope of practice. There were no other differences identified between metropolitan and non-metropolitan community pharmacists in their interest and perceptions regarding pharmacist-prescribed contraception.

## Figures and Tables

**Table 1:** Pharmacist demographics (n = 307).

Characteristic	Total n = 307 n (%)	Community n = 144 n (%)	Non-Community n = 163 n (%)	*p* Value
Gender				
Male	100 (32.6)	64 (44.4)	36 (22.1)	<0.001
Female	207 (67.4)	80 (55.6)	127 (77.9)	
Region				
Metropolitan	252 (82.1)	105 (72.9)	147 (90.2)	<0.001
Nonmetropolitan	55 (17.9)	39 (27.1)	16 (9.8)	
Years in Practice				
1–15	182 (59.3)	76 (52.8)	106 (65.0)	0.06
16–30	74 (24.1)	38 (26.4)	36 (22.1)	
>30	51 (16.6)	30 (20.8)	21 (12.9)	
Age in years				
24–40	168 (54.7)	67 (46.5)	101 (62.0)	0.023
41–55	89 (29.0)	48 (33.3)	41 (25.1)	
>55	50 (16.3)	29 (20.1)	21 (12.9)	

**Table 2:** Community pharmacist educational needs: metropolitan vs. nonmetropolitan.

Education Content	Total n = 144 n (%)	Metropolitan n = 105 n (%)	Non-Metropolitan n = 39 n (%)	*p* Value
Appropriate product selection	114 (79.2)	80 (76.2)	34 (87.2)	0.149
Prescribing for minors	94 (65.3)	68 (64.8)	26 (66.7)	0.831
When to refer to a physician	91 (63.2)	65 (61.9)	26 (66.7)	0.599
Switching between products	89 (61.8)	61 (58.1)	28 (71.8)	0.133
Off-label use	46 (31.9)	33 (31.4)	13 (33.3)	0.828
Contraindications	36 (25.0)	28 (26.7)	8 (20.5)	0.449
Non-contraceptive benefits	36 (25.0)	24 (22.9)	12 (30.8)	0.330
Side effects and possible risks	30 (20.8)	23 (21.9)	7 (17.9)	0.603
Missed dose	21 (14.6)	16 (15.2)	5 (12.8)	0.715
Drug interactions	19 (13.2)	12 (11.4)	7 (17.9)	0.304

**Table 3:** Community-pharmacist-reported potential barriers to pharmacist prescribed contraception.

Potential Barriers	Total n = 144 n (%)	Metropolitan n = 105 n (%)	Non-Metropolitan n = 39 n (%)	*p* Value
Lack of access to patient medical records	101 (70.1)	75 (71.4)	26 (66.7)	0.58
Concern patients will skip preventative visits	94 (65.3)	73 (69.5)	21 (53.8)	0.08
Pharmacist time constraints	92 (63.9)	70 (66.7)	22 (56.4)	0.26
Inadequate compensation	87 (60.4)	65 (61.9)	22 (56.4)	0.55
Increased liabilibity concern	86 (59.7)	65 (61.9)	21 (53.8)	0.38
Resistance from physicians	83 (57.6)	60 (57.1)	23 (59.0)	0.84
Workflow disturbances	74 (51.4)	56 (53.3)	18 (46.2)	0.44
Gaps in my contraceptive knowledge	74 (51.4)	53 (50.5)	21 (53.8)	0.72
Safety concerns	57 (39.6)	44 (41.9)	13 (33.3)	0.35
Lack of private counseling area	34 (23.6)	26 (24.8)	8 (20.5)	0.59

## Data Availability

The data presented in this study are available on request from the corresponding author. The data are not publicly available due to subject privacy.

## Supplementary Materials

The following supporting information can be downloaded at: https://www.mdpi.com/article/10.3390/pharmacy12050156/s1, Figure S1: Pharmacist Survey: Opinions of Pharmacist Prescribed Hormonal Contraception.
