# Supplementary material for: Pharmacist-Prescribed Hormonal Contraception: A Survey of Perceptions of Georgia Community Pharmacists and Non-Community Pharmacists
Source: Pharmacy (Basel). 2024 Oct 18;12(5):156. doi: 10.3390/pharmacy12050156 (PMC11510790; doi:10.3390/pharmacy12050156)
Supplement: Supplementary file 1 [file pharmacy-12-00156-s001.zip › pharmacy-3140453-supplementary.pdf]

Pharmacist Survey: Opinions of Pharmacist Prescribed Hormonal Contraception

UNIVERSITY OF GEORGIA CONSENT LETTER

Dear Participant,

My name is Rebecca Stone and I am a pharmacist and faculty member at the University of Georgia College of Pharmacy. I am inviting you to take part in a research study. Currently pharmacists are able to prescribe hormonal contraception in seventeen states or districts in the US. I am conducting research to identify and document Georgia pharmacists' interest in and concerns regarding pharmacist prescribed hormonal contraception (HC).

I am inviting Pharmacists who are licensed in Georgia to complete a 10 minute survey. Your responses will help understand how pharmacist prescribed contraception is perceived by pharmacists in Georgia.

For your participation, you will receive a $15 Amazon gift card within 2 business days of completing the study. At the end of the survey you will have an opportunity to enter the email address where you will receive the electronic giftcard.

Participation in this survey is voluntary, and completion of the survey is considered consent to participate. You can refuse to take part or stop at any time. Your responses to the survey will be anonymous. The data will be analyzed in aggregate, and reported with descriptive statistics. This research involves the transmission of data over the Internet. Every reasonable effort has been taken to ensure the effective use of available technology; however, as with all internet transmission, confidentiality during online communication cannot be guaranteed.

If you have questions about this research, please feel free to contact me at rhstone@uga.edu. If you have any complaints or questions about your rights as a research volunteer, contact the IRB at 706-542-3199 or by email at IRB@uga.edu. Please keep this letter for your records.

Sincerely,

Rebecca H. Stone, PharmD, BCPS, BCACP, FCCP

Megha D. Patel, MBA, PharmD Candidate

Lara Lindsay, PharmD Candidate

University of Georgia College of Pharmacy

Please provide your Georgia Pharmacist License Number (RPH######)
*This data will only be used to verify you are a pharmacist for gift card receipt. It will not be included in data analysis.

Web address for license lookup if needed: https://gadch.mylicense.com/verification/Search.aspx?facility=N

____

Address on license

____

County on license

____

NCHS code

- ◯ Large central metro
- ◯ Large fringe metro
- ◯ Medium metro
- ◯ Small metro
- ◯ Micropolitan
- ◯ Non-core

Demographic Information

Gender

◯ Male
◯ Female

Age

____

Years in practice

____

Pharmacy Experience

What type of pharmacy practice setting do you work in? (select all that apply)

☐ Retail pharmacy
☐ Independent pharmacy
☐ Health system or hospital pharmacy
☐ Ambulatory care pharmacy
☐ Managed care pharmacy
☐ Pharmaceutical industry
☐ Other (please specify)

Other:

____

Pharmacist Prescribed Hormonal Contraception

If you are interested in an brief update regarding the status of Pharmacist Prescribed Contraception in other states, check out this flyer.

[Attachment: "GA 2021 Update - Pharmacist Fact Sheet.pdf"]

Perspectives regarding Hormonal Contraception (HC)

Are you interested in prescribing hormonal contraception?

◯ Yes
◯ No
◯ Unsure

If no, please briefly share why:

____

If unsure, please briefly share why:

____

Please rate your level of agreement with the following statements:

	Strongly disagree	Disagree	Neutral	Agree	Strongly agree
Pharmacy access to HC (hormonal contraception) would improve patient access and adherence	◯	◯	◯	◯	◯

Provision of HC services is within the pharmacist's scope of practice	○	○	○	○	○
Pharmacy access to HC would be a valuable service for many patients	○	○	○	○	○
Pharmacists are well-trained and educated to prescribe HC	○	○	○	○	○
Provision of HC will result in added responsibility and liability (e.g., requiring malpractice insurance)	○	○	○	○	○
I need additional training to comfortably provide hormonal contraception	○	○	○	○	○
If given the legislative authority today, I would be interested in prescribing hormonal contraceptives in my pharmacy practice	○	○	○	○	○
Being able to independently authorize refills of HC would be helpful in my pharmacy practice	○	○	○	○	○

Please indicate your comfort level in prescribing the following product types:

	Very uncomfortable	Somewhat uncomfortable	Neither comfortable nor uncomfortable	Somewhat comfortable	Very comfortable
Combined Oral Contraception (e.g., Loestrin, Yaz)	○	○	○	○	○
Progestin Only Pill (e.g., Errin)	○	○	○	○	○
Transdermal patch (e.g., Xulane)	○	○	○	○	○
Injection (e.g., Depo Provera)	○	○	○	○	○
Intra-vaginal ring (e.g., NuvaRing, Annovera)	○	○	○	○	○

Which of the following are potential barriers for you to prescribe hormonal contraceptives? Select all that apply.

- ☐ Inadequate compensation
- ☐ Workflow disturbances
- ☐ Time constraints of pharmacists
- ☐ Lack of private counseling area
- ☐ Safety concerns
- ☐ Resistance from physicians
- ☐ Lack of access to patient medical records
- ☐ Concern that patients will skip women's health practitioner visits
- ☐ Increased liability concern
- ☐ Gaps in my contraceptive knowledge

If you were to offer this service in your pharmacy, which of the following additional resources would you need? Select all that apply.

- ☐ More staffing
- ☐ Reimbursement for time (i.e. consult visit fee paid by patient or insurance)
- ☐ Private screening areas
- ☐ Access to electronic medical records
- ☐ Other

describe other

____

If provided with appropriate continuing education, choose the statement that reflects your confidence in the ability to:

	Not at all confident	Moderately confident	Very confident
Use a patient screening tool and blood pressure reading to determine eligibility for hormonal contraception based on the U.S. Medical Eligibility Criteria for Contraceptive Use	○	○	○
Use patient-specific factors (i.e., PMH, medication history, preference, etc.) to choose an appropriate hormonal contraceptive product	○	○	○

Are there specific aspects of hormonal contraception use where you would need more education? (Select all that apply)

- ☐ Appropriate product selection
- ☐ Switching between products
- ☐ Off-label use
- ☐ Drug interactions
- ☐ Counseling on contraceptive mishaps (ie missed dose)
- ☐ Side effects and possible risks
- ☐ When to refer to a physician
- ☐ Contraindications
- ☐ Non-contraceptive benefits of hormonal contraception
- ☐ Prescribing for minors
- ☐ I feel confident providing all these services without additional education
- ☐ I do not intend to prescribe hormonal contraception

Perspectives regarding emergency contraception (EC)

	Disagree	Neutral	Agree
I feel comfortable instructing patients about appropriate use of the emergency contraceptive Plan B (levonorgestrel)	○	○	○

I feel comfortable instructing patients about appropriate use of the emergency contraceptive ella (ulipristal acetate)	○	○	○
I am uncomfortable dispensing EC for moral or religious reasons	○	○	○

If you would like to receive a $15 Amazon gift card, please enter your preferred email address here: __________________________

*Gift cards will be emailed within 2 business days of survey completion - watch your email!

**Your survey answers will remain anonymous and confidential
